# Novel HPAIV H5N8 Reassortant (Clade 2.3.4.4b) Detected in Germany

**DOI:** 10.3390/v12030281

**Published:** 2020-03-04

**Authors:** Jacqueline King, Christoph Schulze, Andreas Engelhardt, Andreas Hlinak, Sara-Lisa Lennermann, Kerstin Rigbers, Jasmin Skuballa, Christoph Staubach, Thomas C. Mettenleiter, Timm Harder, Martin Beer, Anne Pohlmann

**Affiliations:** 1 Institute of Diagnostic Virology, Friedrich-Loeffler-Institut, 17493 Greifswald–Insel Riems, Germany; jacqueline.king@fli.de (J.K.); anne.pohlmann@fli.de (A.P.); 2 Landeslabor Berlin-Brandenburg (LLBB), 15236 Frankfurt (Oder), Germany; christoph.schulze@landeslabor-bbb.de (C.S.); andreas.engelhardt@landeslabor-bbb.de (A.E.); andreas.hlinak@landeslabor-bbb.de (A.H.); 3 Chemisches und Veterinäruntersuchungsamt Karlsruhe, 76187 Karlsruhe, Germany; 4 Institute of Epidemiology, Friedrich-Loeffler-Institut, 17493 Greifswald, Insel Riems, Germany; christoph.staubach@fli.de; 5 President, Friedrich-Loeffler-Institut, 17493 Greifswald, Insel Riems, Germany; thomas.mettenleiter@fli.de

**Keywords:** avian influenza viruses, HPAIV, reassortment, H5N8, third-generation sequencing, MinION

## Abstract

A novel H5N8 highly pathogenic avian influenza virus (HPAIV) was detected in a greater white-fronted goose in January 2020 in Brandenburg, Germany, and, in February 2020, in domestic chickens belonging to a smallholding in Baden-Wuerttemberg, Germany. Full-genome sequencing was conducted on the MinION platform, enabling further phylogenetic analyses. The virus of clade 2.3.4.4b holds six segments from a Eurasian/Asian/African HPAIV H5N8 reassortant and two segments from low pathogenic avian influenza H3N8 subtype viruses recently detected in wild birds in Central Russia. These new entries continue to show the reassortment potential of the clade 2.3.4.4 H5Nx viruses, underlining the necessity for full-genome sequencing and continuous surveillance.

## 1. Introduction

The severe European epizootic of highly pathogenic avian influenza viruses (HPAIV), peaking during the winter of 2016–2017, was dominated by viral swarm incursions and frequent reassortment events [1]. All belonged to group B of clade 2.3.4.4, six reassortants classifying into three subtypes were identified in Germany from November 2016 to August 2018 [2,3,4]. Phylogenetic analyses of the H5N8 subtypes pointed to individual incursion events, as similar H5N8 HPAIV reassortants were found prior to the German epizootic in migratory wild water bird molting and resting areas in the regions surrounding Tartastan, Kurgan, and Lake Chany, Russian Federation [2,5]. The pronounced magnitude and economic impact of this outbreak attested to the eminent pathogenicity of clade 2.3.4.4b HPAIV [6]. Starting in December 2019, several reports of HPAI H5N8 cases in central and eastern Europe were broadcasted from the responsible national authorities (OIE; https://www.oie.int/en/animal-health-in-the-world/update-on-avian-influenza/2020/). Since January 2020, a clade 2.3.4.4b virus has also been detected in Germany in the form of a novel H5N8 reassortant, Ger-01-20.

## 2. Materials and Methods

**Figure 1:**
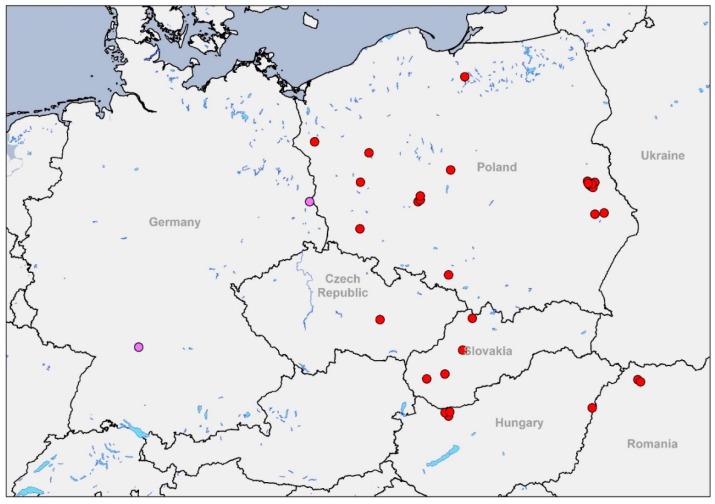
Geographical distribution of European HPAIV H5N8 detections from December 2019 to February 2020 (status as of 13 February 2020). The two German cases are highlighted in purple. Maps were plotted using data from the OIE website and the German animal disease notification system (TSN).

On 16 January 2020, a greater white-fronted goose (*Anser albifrons*) was found dead close to the Polish border in the federal state of Brandenburg, Germany (Figure 1). Pathological examination determined trauma as a cause of death; nevertheless, initial testing for avian influenza virus RNA revealed very high virus loads in mixed tissue homogenates (lung and gut tissue) with RT–qPCR Cq-values of RT–qPCR H5 Cq = 16.1 and RT–qPCR N8.2 Cq = 14.1. Primary sub- and pathotyping results achieved via qPCR [7] revealed a HPAIV H5N8 of clade 2.3.4.4b (RT–qPCR H5 HP 2344b DE Cq = 14.1). Subsequently, a severe necrotizing polioencephalitis, typical of H5N8 infection in waterfowl [8], was detected by histopathology, most likely causing disorientation and predisposing the goose to the traumatic event.

Shortly after, on 6 February 2020, chickens (*Gallus gallus domesticus*) from a small holding in the federal state of Baden-Wuerttemberg (Figure 1) also tested positive for HPAIV H5N8 of clade 2.3.4.4b (RT–qPCR H5 HP 2344b DE Cq = 22.9–25.9; RT–qPCR N8.2 Cq = 22.2–25.4) following the same testing protocol as described [7]. In this case, RNA was extracted from swab samples. During necropsy, the birds showed moderate mucous discharge in the upper respiratory tract and diarrhea. In addition, a severe acute diffuse necrotizing lymphohistiocytic enteritis and a moderate necrotizing encephalitis with perivascular cuffing and gliosis were determined as major characteristic histological lesions.

Amplification for MinION-assisted full genome sequencing of the RNA from both outbreaks was conducted prior to sequencing utilizing the Superscript III One-Step AIV-End-RT-PCR with Platinum Taq (ThermoFisher Scientific, Waltham, MA, USA) and universal AIV primers designed for the conserved ends of all segments [9]. Subsequently, after library preparation with the Rapid Barcoding Kit (RBK-004, Oxford Nanopore Technology, Oxford, UK; ONT) according to the manufacturer’s instructions, full genome sequencing was performed on the MinION platform in combination with a R9.4 flow cell (ONT), the MinIT (v19.12.1; ONT) and basecaller Guppy (v3.2.9; ONT), facilitating real-time basecalling to produce quality checked, demultiplexed, and trimmed raw data.

Consensus assembly of the sequencing data was executed with the Geneious Prime software (Biomatters, Auckland, New Zealand) in a map to reference approach, while further phylogenetic analyses were completed with RAxML [10] and SplitsTree4 [11].

Full genome sequences were deposited into the GISAID database (www.gisaid.org) under the accession numbers EPI_ISL_404993 (2020AI00018; A/white-fronted goose/Germany-BB/AI00018/2020) and EPI_ISL_410291 (2020AI00049; A/chicken/Germany-BW/AI00049/2020). Further genome sequences acquired from the GISAID database and utilized for phylogenetic analyses have been acknowledged in Table S2.

## 3. Results

**Figure 2:**
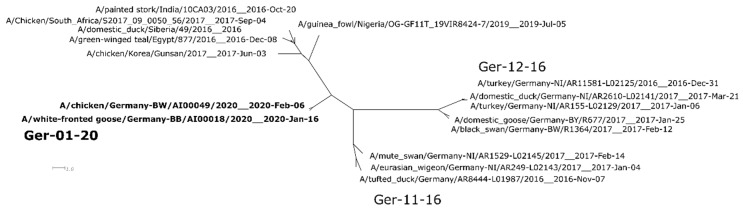
Supernetwork of full genomes of selected worldwide clade 2.3.4.4b H5N8 subgroups from maximum likelihood (ML) trees of PB2, PB1, PA, HA, NP, NA, MP, and NS segments. ML analyses were done using RAxML, including bootstrapping for 1000 iterations and network analyses conducted with SplitsTree4.

Analyses of the full genome sequences from both outbreaks allowed for the identification of a novel reassortant, Ger-01-20, revealing a distinct segment combination that differs from reassortants described in Germany 2016/2017 and similar reassortants circulating worldwide (Figure 2).

In comparison to the HPAIVs circulating during the 2016–2018 epizootic [2,5], the Ger-01-20 reassortant comprises of eight unique segments newly introduced to Germany (Figure 2 and Figure S1).

Six of the eight segments (1, 3, 4, 6, 7, and 8) cluster with 98% sequence identity to a previous HPAIV H5N8 reassortant found from 2016 onwards (Figure 2 and Figure S1, Table S1) [12,13]. This reassortant has been identified in areas ranging from Asia/Eurasia (India, A/painted stork/India/10CA03/2016; South Korea, A/common teal/Korea/W548_2016, A/chicken/Korea/Gunsan/2017; Central Russia, Siberia, A/domestic duck/Siberia/49/2016), Europe (Italy, A/turkey /Italy/17VIR538-1/2017) [14] to Africa (Egypt, A/green-winged teal/Egypt/877/2016; South Africa, A/Geese/South_Africa/S2017/09_0055_P1/2017) in the season 2016/2017 [12,15]. On closer examina-tion, the segments 1, 3, 4, 7, and 8 also exhibit high identity levels (98.86%–99.55%, Table S1) to a recent Nigerian HPAIV H5N8 (A/guinea fowl/Nigeria/OG-GF11T_19VIR8724-7) sampled in July 2019, while segment 6 has proven to share the highest sequence identity with HPAI H5N8 viruses circulating in Siberia, India, and Korea from 2016 to 2017 (98.56%–98.49%, Table S1).

**Figure 3:**
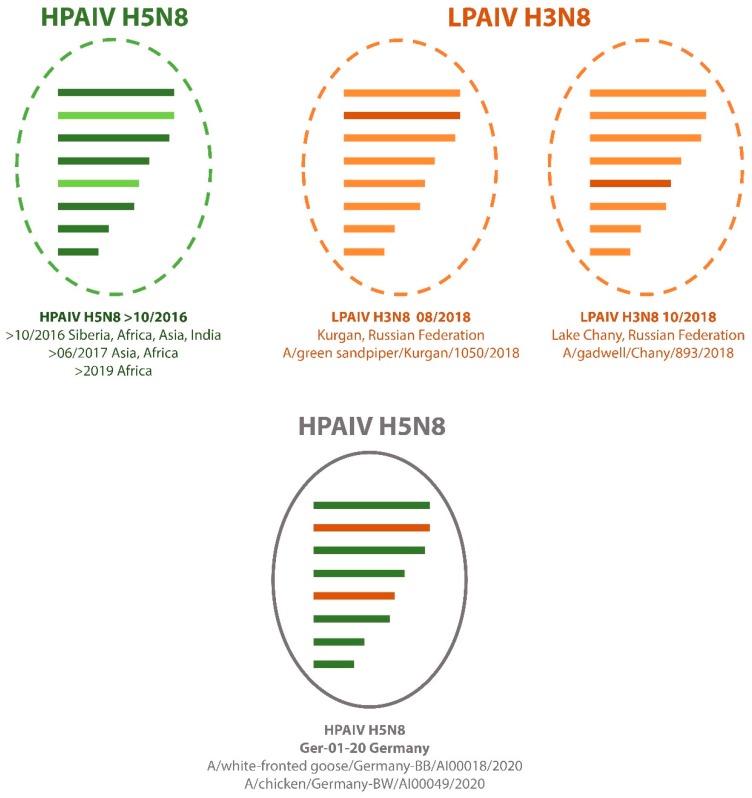
Schematic reassortment analyses based on full-length sequences from AIVs previously detected (upper panel) and the novel HPAIV H5N8 reassortant Ger-01-20 (lower panel). Colors are allocated according to reassortment and display the new composition of HPAIV H5N8 Ger-01-20.

The segments PB1 and NP, respectively, display high similarities to a low pathogenic avian influenza virus (LPAIV) of the subtype H3N8 found in wild waterbirds located in Central Russia. Segment 2 was proven to share 99.01% identity (Table S1) to a sequence from a green sandpiper from the Kurgan area of Central Russia (A/green sandpiper/Kurgan/1050/2018) sampled in late August 2018. Along these lines, segment 5 showed comparable identity levels (98.51%, Table S1) to an LPAIV from a gadwall found at Lake Chany, Central Russia (A/gadwall/Chany/893/2018), sampled shortly after in mid-October 2018 (Figure 3 and Figure S1).

Overall, the phylogenetic analyses demonstrate high similarity ranging from 99.53%–100% (Table S1) between the novel HPAIV H5N8 Ger-01-20 reassortant detected in both a wild bird and poultry and newly released sequences from Poland (A/turkey/Poland/23/2019 and A/hawk/Poland/003/2020) and the Czech Republic (A/chicken/Czech_Republic/1175-1/2020; Figure S1). Further investigation indicates a clustering of the virus from the greater white-fronted goose found in Brandenburg with both Polish sequences while the poultry outbreak in Baden-Wuerttemberg displays higher genetic similarities to the Czech Republic case (Figure S1).

## 4. Discussion

HP viruses of the gs/GD lineage of clade 2.3.4.4b are reportedly highly capable in attaining novel genome segments through reassortment events, a significant distinction to other gs/GD clades such as Egyptian 2.2.1.x viruses known for their genotypic stability for over more than a decade [16,17]. Promiscuity with respect to reassortment is expected to translate into potentially advantageous phenotypic features affecting viral host range and fitness. This is mirrored by the continuous and unprecedented spread of clade 2.3.4.4 viruses represented by more than 25 reassorted genotypes across Asia, Northern America, Europe, and Africa since 2014 [16].

Based on the genetic composition of the novel HPAIV H5N8 reassortant Ger-01-20, a new incursion event into Germany is highly likely and no direct genetic correlation to German H5N8 subtypes circulating during the 2016 to 2017 epizootic was established. Currently, no distinct precursor for the Ger-01-20 reassortant has been identified. The role of migratory waterbirds in the distribution of AIVs, as a catalyst for reassortment events and as the natural reservoir for LPAIV, has been demonstrated by numerous studies [3,18,19,20]. Harboring of segments with sequences related to those identified in an H3N8 LPAIV in wild waterbirds from Central Russia along with segments from the HPAIV H5N8 reassortant found in Eurasia and Africa also infers a connection between the novel reassortant and migratory birds from the African Eurasian flyway as well as molting/resting grounds along the Russian–Kazakhstan/Mongolian/Chinese border [19].

The identification of the same reassortant in multiple central and eastern European countries indicates continuous circulation of the virus and demonstrates genetic connections between these cases and both German outbreaks. As a result, in combination with the notification dates and geographical locations broadcasted by the responsible national authorities (OIE; https://www.oie.int/en/animal-health-in-the-world/update-on-avian-influenza/2020/), it seems permissible to speculate an incursion into Germany from the East. Although genetic relations of Ger-01-20 with numerous HPAI H5N8 viruses found on the African continent (e.g., Nigeria, South Africa, Egypt) could be determined, the connection to LPAIVs found only in central Russia indicates that the African viruses are unlikely to be direct precursors, but instead suggests the circulation of similar HPAIVs on the African continent, possibly sharing the same ancestor.

Based on the limited information available at this time, no clear conclusions as to the circulation of this novel reassortant in wild birds can be drawn. The described German wild bird case is only the second of its kind in Europe in this season, with the first infection identified in a goshawk found in close proximity to an affected poultry holding in Poland. However, the identification of the novel reassortant virus in both wild birds and poultry points towards their susceptibility for infection. Thus, in addition to infected poultry, wild birds must be regarded as a reservoir and vector of this HPAIV.

## 5. Conclusions

The detection of the new H5N8 subtype Ger-01-20 reassortant, with its genetic backbone reverting to clade 2.3.4.4b, displays the continuing circulation of this clade and highlights its tendency for frequent reassortments and efficient long-distance transmissions. On closer inspection, the virus consists of six segments from the Eurasian/Asian/African HPAIV H5N8 reassortant and two segments from a LPAIV H3N8 subtype found in central Russia. Both German outbreaks show related genetic constellations to sequence data from Poland and the Czech Republic sampled from December 2019 to January 2020. These findings emphasize the necessity for full-genome sequencing and continuous passive surveillance in order to rapidly detect and identify novel HPAIVs, even more so due to the unprecedented genetic variety this clade entails.

## Supplementary Materials

The following are available online at https://www.mdpi.com/1999-4915/12/3/281/s1, Figure S1: Phylogenetic analyses of Ger-01-20, Table S1: Sequence identity (%), Table S2: Data acknowledgment.
