# Supplementary material for: Novel HPAIV H5N8 Reassortant (Clade 2.3.4.4b) Detected in Germany
Source: Viruses. 2020 Mar 4;12(3):281. doi: 10.3390/v12030281 (PMC7150876; doi:10.3390/v12030281)
Supplement: Supplementary file 1 [file viruses-12-00281-s001.pdf]

Supplementary Materials: Novel H5N8 Reassortant (clade 2.3.4.4b) detected in Germany

Figure S1: Phylogenetic analyses of PB2, PB1, PA, HA, NP, NA, MP and NS sequences done by Maximum Likelihood trees using RAxML with a bootstrap value of 1000 cycles. Trees include the novel reassortant Ger-01-20 (A/white-fronted goose/Germany-BB/AI00018/2020 (root) and A/chicken/Germany-BW/AI00049/2020 – both in blue lettering), and 100 closest relatives according to sequence identity. The red segments cluster with the Ger-01-20 reassortant, all originating in Eastern Europe (Poland and the Czech Republic) from December 2019 – January 2020. The dark green segments pinpoint the HPAIV H5N8 >10-2016 (Nigeria, Korea, India, South Africa) reassortant, while the orange marked sequences stand for the LPAIV H3N8 (Russia, >Aug 2017).

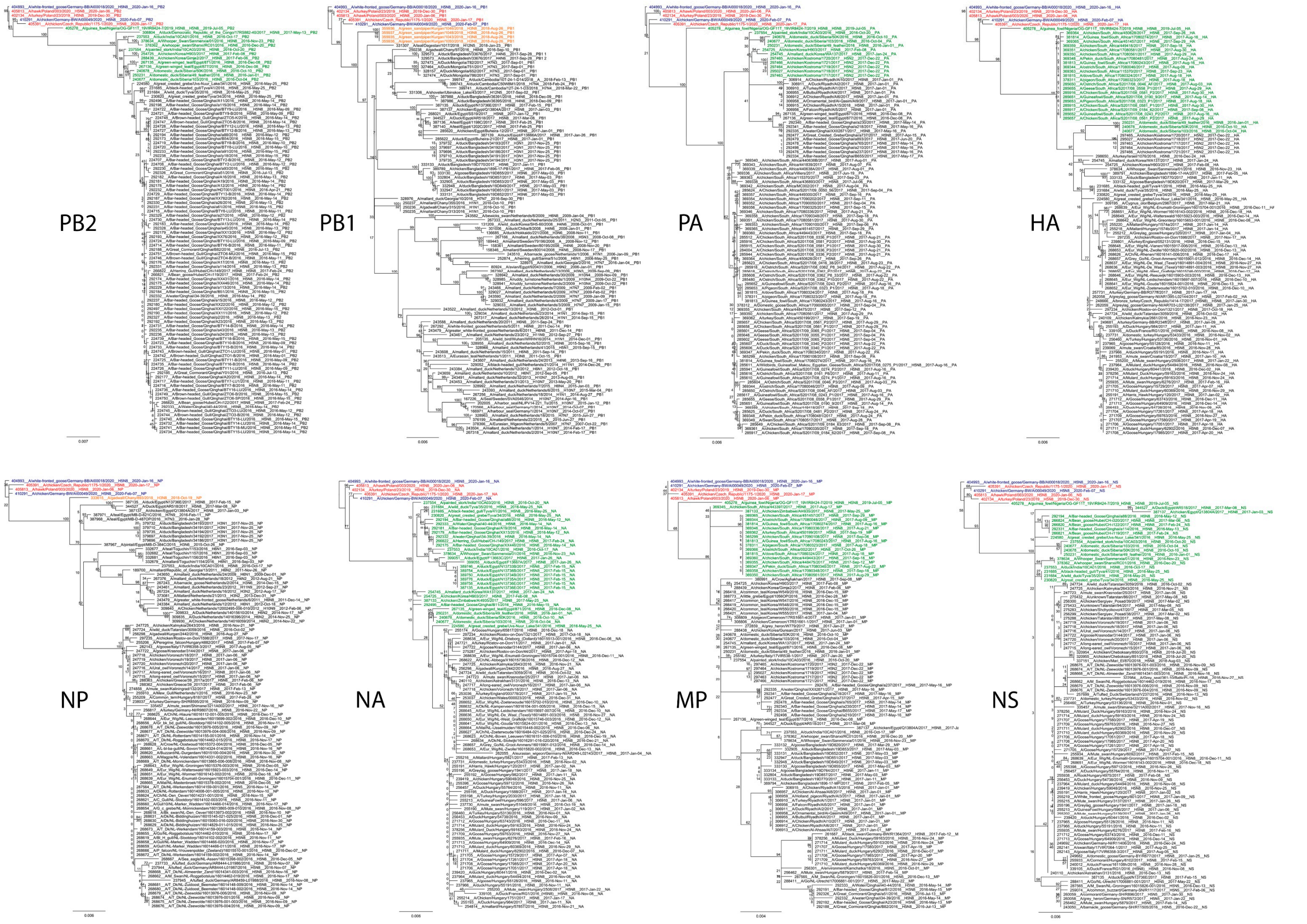

Table S1: Sequence identity (%) of A/white-fronted goose/Germany-BB/AI00018/2020 in comparison to the top ten Blast results (executed on the GISAID Platform – 13.02.2020).

	ID	Name	Subtype	Collection Date	Sequence Identity %
**PB2**	EPI_ISL_405813	A/hawk/Poland/003/2020	H5N8	2020-Jan-06	99.96
	EPI_ISL_402134	A/turkey/Poland/23/2019	H5N8	2019-Dec-30	99.93
	EPI_ISL_410291	A/chicken/Germany-BW/AI00049/2020	H5N8	2020-Feb-07	99.78
	EPI_ISL_405391	A/chicken/Czech_Republic/1175-1/2020	H5N8	2020-Jan-17	99.61
	EPI_ISL_405278	A/guinea_fowl/Nigeria/OG-GF11T_19VIR8424-7/2019	H5N8	2019-Jul-05	98.86
	EPI_ISL_237554	A/painted_stork/India/10CA03/2016	H5N8	2016-Oct-20	97.65
	EPI_ISL_240677	A/domestic_duck/Siberia/103/2016	H5N8	2016-Oct-04	97.58
	EPI_ISL_250231	A/domestic_duck/Siberia/49_feather/2016	H5N8	2016-Jan-01	97.58
	EPI_ISL_267135	A/green-winged_teal/Egypt/871/2016	H5N8	2016-Dec-08	97.56
	EPI_ISL_224580	A/great_crested_grebe/Uvs-Nuur_Lake/341/2016	H5N8	2016-May-25	97.50
	EPI_ISL_237553	A/duck/India/10CA01/2016	H5N8	2016-Oct-17	97.50
**PB1**	EPI_ISL_402134	A/turkey/Poland/23/2019	H5N8	2019-Dec-30	99.83
	EPI_ISL_405813	A/hawk/Poland/003/2020	H5N8	2020-Jan-06	99.82
	EPI_ISL_405391	A/chicken/Czech_Republic/1175-1/2020	H5N8	2020-Jan-17	99.61
	EPI_ISL_410291	A/chicken/Germany-BW/AI00049/2020	H5N8	2020-Feb-07	99.46
	EPI_ISL_355938	A/green_sandpiper/Kurgan/1050/2018	H3N8	2018-Aug-26	99.01
	EPI_ISL_331307	A/teal/Dagestan/1017/2018	H12N5	2018-Jan-23	98.72
	EPI_ISL_250237	A/mallard/Chany/355/2016	H1N1	2016-Oct-10	98.71
	EPI_ISL_250236	A/gadwall/Chany/315/2016	H1N1	2016-Oct-10	98.71
	EPI_ISL_250235	A/mallard/Chany/313/2016	H1N1	2016-Oct-10	98.71
	EPI_ISL_328976	A/mallard_duck/Georgia/10/2016	H7N7	2016-Sep-30	98.66
	EPI_ISL_250238	A/gadwall/Chany/97/2016	H6N8	2016-Sep-10	98.62
**PA**	EPI_ISL_410291	A/chicken/Germany-BW/AI00049/2020	H5N8	2020-Feb-07	99.77
	EPI_ISL_405813	A/hawk/Poland/003/2020	H5N8	2020-Jan-06	99.72
	EPI_ISL_402134	A/turkey/Poland/23/2019	H5N8	2019-Dec-30	99.72
	EPI_ISL_405391	A/chicken/Czech_Republic/1175-1/2020	H5N8	2020-Jan-17	99.55
	EPI_ISL_405278	A/guinea_fowl/Nigeria/OG-GF11T_19VIR8424-7/2019	H5N8	2019-Jul-05	98.98
	EPI_ISL_378312	A/Domestic_goose/South_Africa/17090065/2017	H5N8	2017-Sep-04	98.51
	EPI_ISL_369355	A/chicken/South_Africa/448475/2017	H5N8	2017-Sep-12	98.51

	EPI_ISL_369339	A/chicken/South_Africa/440638A/2017	H5N8	2017-Aug-01	98.51
	EPI_ISL_369336	A/chicken/South_Africa/Villiers/2017	H5N8	2017-Jun-19	98.51
	EPI_ISL_285657	A/Chicken/South_Africa/S2017/08_0561_P2/2017	H5N8	2017-Aug-29	98.50
	EPI_ISL_285648	A/Geese/South_Africa/S2017/09_0055_P1/2017	H5N8	2017-Sep-04	98.50
**HA**	EPI_ISL_405813	A/hawk/Poland/003/2020	H5N8	2020-Jan-06	100.00
	EPI_ISL_402134	A/turkey/Poland/23/2019	H5N8	2019-Dec-30	100.00
	EPI_ISL_410291	A/chicken/Germany-BW/AI00049/2020	H5N8	2020-Feb-07	99.60
	EPI_ISL_405391	A/chicken/Czech_Republic/1175-1/2020	H5N8	2020-Jan-17	99.54
	EPI_ISL_405278	A/guinea_fowl/Nigeria/OG-GF11T_19VIR8424-7/2019	H5N8	2019-Jul-05	99.06
	EPI_ISL_381815	A/dove/South_Africa/17080324/2017	H5N8	2017-Aug-18	98.42
	EPI_ISL_381813	A/Guinea_fowl/South_Africa/17080243/2017	H5N8	2017-Aug-16	98.42
	EPI_ISL_378311	A/pigeon/South_Africa/17080323/2017	H5N8	2017-Aug-18	98.42
	EPI_ISL_369359	A/chicken/South_Africa/449418/2017	H5N8	2017-Sep-18	98.42
	EPI_ISL_369351	A/chicken/South_Africa/17080581/2017	H5N8	2017-Aug-30	98.42
	EPI_ISL_369348	A/Pekin_duck/South_Africa/17080481/2017	H5N8	2017-Aug-24	98.42
**NP**	EPI_ISL_410291	A/chicken/Germany-BW/AI00049/2020	H5N8	2020-Feb-07	99.81
	EPI_ISL_405813	A/hawk/Poland/003/2020	H5N8	2020-Jan-06	99.79
	EPI_ISL_405391	A/chicken/Czech_Republic/1175-1/2020	H5N8	2020-Jan-17	99.57
	EPI_ISL_387968	A/teal/Egypt/MB-D-487OP/2016	H7N3	2016-Jan-28	98.51
	EPI_ISL_333615	A/gadwall/Chany/893/2018	H3N8	2018-Oct-19	98.51
	EPI_ISL_387971	A/teal/Egypt/MB-D-621C/2016	H7N9	2016-Feb-11	98.44
	EPI_ISL_387967	A/pintail/Egypt/MB-D-384C/2015	H3N6	2015-Oct-26	98.25
	EPI_ISL_387135	A/duck/Egypt/N13736E/2017	H5N8	2017-Feb-15	97.99
	EPI_ISL_344527	A/Duck/Egypt/AR518/2017	H5N8	2017-Mar-08	97.99
	EPI_ISL_387137	A/chicken/Egypt/Q13804A/2017	H5N8	2017-Jan-03	97.92
	EPI_ISL_189700	A/mallard/Republic_of_Georgia/13/2011	H6N2	2011-Nov-26	97.84
**NA**	EPI_ISL_402134	A/turkey/Poland/23/2019	H5N8	2019-Dec-30	99.68
	EPI_ISL_405813	A/hawk/Poland/003/2020	H5N8	2020-Jan-06	99.58
	EPI_ISL_410291	A/chicken/Germany-BW/AI00049/2020	H5N8	2020-Feb-07	99.55
	EPI_ISL_405391	A/chicken/Czech_Republic/1175-1/2020	H5N8	2020-Jan-17	99.47
	EPI_ISL_250231	A/domestic_duck/Siberia/49_feather/2016	H5N8	2016-Jan-01	98.56
	EPI_ISL_237554	A/painted_stork/India/10CA03/2016	H5N8	2016-Oct-20	98.50

	EPI_ISL_254725	A/chicken/Korea/H903/2017	H5N8	2017-Feb-08	98.49
	EPI_ISL_387133	A/chicken/Zimbabwe/AI4935/2017	H5N8	2017-May-25	98.48
	EPI_ISL_292495	A/Bar-headed_Goose/Qinghai/B11/2016	H5N8	2016-May-15	98.48
	EPI_ISL_240677	A/domestic_duck/Siberia/103/2016	H5N8	2016-Oct-04	98.43
**MP**	EPI_ISL_410291	A/chicken/Germany-BW/AI00049/2020	H5N8	2020-Feb-07	100.00
	EPI_ISL_405813	A/hawk/Poland/003/2020	H5N8	2020-Jan-06	100.00
	EPI_ISL_402134	A/turkey/Poland/23/2019	H5N8	2019-Dec-30	100.00
	EPI_ISL_405391	A/chicken/Czech_Republic/1175-1/2020	H5N8	2020-Jan-17	99.90
	EPI_ISL_369345	A/chicken/South_Africa/443397/2017	H5N8	2017-Aug-17	99.59
	EPI_ISL_267135	A/green-winged_teal/Egypt/871/2016	H5N8	2016-Dec-08	99.50
	EPI_ISL_250231	A/domestic_duck/Siberia/49_feather/2016	H5N8	2016-Jan-01	99.50
	EPI_ISL_240677	A/domestic_duck/Siberia/103/2016	H5N8	2016-Oct-04	99.50
	EPI_ISL_254745	A/mallard_duck/Korea/WA137/2017	H5N8	2017-Jan-24	99.50
	EPI_ISL_388773	A/little_grebe/Egypt/1056OP/2016	H5N8	2016-Dec-28	99.49
	EPI_ISL_266421	A/common_teal/Korea/W555/2017	H5N8	2017-Jan-04	99.49
**NS**	EPI_ISL_410291	A/chicken/Germany-BW/AI00049/2020	H5N8	2020-Feb-07	99.83
	EPI_ISL_405813	A/hawk/Poland/003/2020	H5N8	2020-Jan-06	99.76
	EPI_ISL_402134	A/turkey/Poland/23/2019	H5N8	2019-Dec-30	99.64
	EPI_ISL_405391	A/chicken/Czech_Republic/1175-1/2020	H5N8	2020-Jan-17	99.53
	EPI_ISL_405278	A/guinea_fowl/Nigeria/OG-GF11T_19VIR8424-7/2019	H5N8	2019-Jul-05	99.16
	EPI_ISL_237553	A/duck/India/10CA01/2016	H5N8	2016-Oct-17	98.61
	EPI_ISL_231685	A/black-headed_gull/Tyva/41/2016	H5N8	2016-May-25	98.61
	EPI_ISL_231684	A/wild_duck/Tyva/35/2016	H5N8	2016-May-25	98.61
	EPI_ISL_230820	A/great_crested_grebe/Tyva/34/2016	H5N8	2016-May-25	98.61
	EPI_ISL_292194	A/Bar-headed_Goose/Qinghai/a88/2016	H5N8	2016-May-12	98.60
	EPI_ISL_266820	A/Bean_goose/Hubei/CH-i122/2017	H5N8	2017-Feb-24	98.60

Table S2: Data acknowledgement – We acknowledge the following laboratories for providing sequences in the EpiFlu™ Database.

Isolate ID	Isolate Name	Submitting Lab
EPI_ISL_355938	A/green_sandpiper/Kurgan/1050/2018	State Research Center of Virology and Biotechnology
EPI_ISL_355937	A/green_sandpiper/Kurgan/1048/2018	State Research Center of Virology and Biotechnology
EPI_ISL_355936	A/green_sandpiper/Kurgan/1046/2018	State Research Center of Virology and Biotechnology
EPI_ISL_355935	A/green_sandpiper/Kurgan/1043/2018	State Research Center of Virology and Biotechnology
EPI_ISL_250237	A/mallard/Chany/355/2016	Research Institute of Experimental and Clinical Medicine
EPI_ISL_250236	A/gadwall/Chany/315/2016	Research Institute of Experimental and Clinical Medicine
EPI_ISL_250235	A/mallard/Chany/313/2016	Research Institute of Experimental and Clinical Medicine
EPI_ISL_250238	A/gadwall/Chany/97/2016	Research Institute of Experimental and Clinical Medicine
EPI_ISL_331306	A/shoveler/Ubinskoe_Lake/43/2017	Research Institute of Experimental and Clinical Medicine
EPI_ISL_331307	A/teal/Dagestan/1017/2018	Research Institute of Experimental and Clinical Medicine
EPI_ISL_167226	A/Seal/Sweden/SVA0546/2014	National Veterinary Institute, Uppsala, Sweden
EPI_ISL_285657	A/Chicken/South_Africa/S2017/08_0561_P2/2017	National Institute for Communicable Diseases
EPI_ISL_285648	A/Geese/South_Africa/S2017/09_0055_P1/2017	National Institute for Communicable Diseases
EPI_ISL_378312	A/Domestic_goose/South_Africa/17090065/2017	National Institute for Communicable Diseases
EPI_ISL_369355	A/chicken/South_Africa/448475/2017	National Institute for Communicable Diseases
EPI_ISL_285601	A/Geese/South_Africa/S2017/09_0065_P1/2017	National Institute for Communicable Diseases
EPI_ISL_285611	A/Wildbirds_Guineafowl_Makou_Egyptian_Geese/South_Africa/S2017/08_0275_P1/2017	National Institute for Communicable Diseases
EPI_ISL_381814	A/Guinea_fowl/South_Africa/17080274/2017	National Institute for Communicable Diseases
EPI_ISL_285941	A/Guineafowl/South_Africa/S2017/08_0274_P2/2017	National Institute for Communicable Diseases
EPI_ISL_369350	A/chicken/South_Africa/17080561/2017	National Institute for Communicable Diseases
EPI_ISL_285607	A/Duck/South_Africa/S2017/08_0340_P2/2017	National Institute for Communicable Diseases
EPI_ISL_285606	A/Duck/South_Africa/S2017/08_0340_P1/2017	National Institute for Communicable Diseases
EPI_ISL_369347	A/Pekin_duck/South_Africa/17080340/2017	National Institute for Communicable Diseases
EPI_ISL_365299	A/chicken/South_Africa/17090108/2017	National Institute for Communicable Diseases
EPI_ISL_285651	A/Guineafowl/South_Africa/S2017/08_0190_9/2017	National Institute for Communicable Diseases
EPI_ISL_369349	A/Swan/South_Africa/17080517/2017	National Institute for Communicable Diseases
EPI_ISL_369348	A/Pekin_duck/South_Africa/17080481/2017	National Institute for Communicable Diseases
EPI_ISL_369361	A/chicken/South_Africa/17090335/2017	National Institute for Communicable Diseases
EPI_ISL_285610	A/Guineafowl/South_Africa/S2017/08_0274_P1/2017	National Institute for Communicable Diseases
EPI_ISL_285916	A/Chicken/South_Africa/S2017/08_0581_P2/2017	National Institute for Communicable Diseases
EPI_ISL_369351	A/chicken/South_Africa/17080581/2017	National Institute for Communicable Diseases
EPI_ISL_369346	A/chicken/South_Africa/17080336/2017	National Institute for Communicable Diseases
EPI_ISL_285624	A/Chicken/South_Africa/S2017/09_0050_56/2017	National Institute for Communicable Diseases
EPI_ISL_369357	A/chicken/South_Africa/449300/2017	National Institute for Communicable Diseases
EPI_ISL_369356	A/chicken/South_Africa/17090325/2017	National Institute for Communicable Diseases
EPI_ISL_369354	A/chicken/South_Africa/17090202/2017	National Institute for Communicable Diseases
EPI_ISL_369352	A/chicken/South_Africa/17090050/2017	National Institute for Communicable Diseases
EPI_ISL_285652	A/Guineafowl/South_Africa/S2017/08_0243_P2/2017	National Institute for Communicable Diseases
EPI_ISL_381815	A/dove/South_Africa/17080324/2017	National Institute for Communicable Diseases
EPI_ISL_381813	A/Guinea_fowl/South_Africa/17080243/2017	National Institute for Communicable Diseases
EPI_ISL_378311	A/pigeon/South_Africa/17080323/2017	National Institute for Communicable Diseases
EPI_ISL_369360	A/chicken/South_Africa/449443/2017	National Institute for Communicable Diseases

EPI_ISL_369353	A/chicken/South_Africa/17090100/2017	National Institute for Communicable Diseases
EPI_ISL_284004	A/Chicken/South_Africa/S2017/08_0336_P3/2017	National Institute for Communicable Diseases
EPI_ISL_369364	A/chicken/South_Africa/450628/2017	National Institute for Communicable Diseases
EPI_ISL_285623	A/Chicken/South_Africa/S2017/08_0416_38/2017	National Institute for Communicable Diseases
EPI_ISL_369358	A/chicken/South_Africa/17090348/2017	National Institute for Communicable Diseases
EPI_ISL_369365	A/chicken/South_Africa/451457/2017	National Institute for Communicable Diseases
EPI_ISL_285915	A/Chicken/South_Africa/S2017/08_0581_P1/2017	National Institute for Communicable Diseases
EPI_ISL_285655	A/Geese/South_Africa/S2017/08_0558_P1/2017	National Institute for Communicable Diseases
EPI_ISL_369359	A/chicken/South_Africa/449418/2017	National Institute for Communicable Diseases
EPI_ISL_285617	A/Guineafowl/South_Africa/S2017/08_0243_P1/2017	National Institute for Communicable Diseases
EPI_ISL_285658	A/Chicken/South_Africa/S2017/08_0561_P1/2017	National Institute for Communicable Diseases
EPI_ISL_285650	A/Ostrich/South_Africa/S2017/08_0046_AF/2017	National Institute for Communicable Diseases
EPI_ISL_369344	A/ostrich/South_Africa/17080046/2017	National Institute for Communicable Diseases
EPI_ISL_369362	A/turkey/South_Africa/450199/2017	National Institute for Communicable Diseases
EPI_ISL_285649	A/Chicken/South_Africa/S2017/09_0184_63/2017	National Institute for Communicable Diseases
EPI_ISL_285602	A/Geese/South_Africa/S2017/09_0065_P2/2017	National Institute for Communicable Diseases
EPI_ISL_285918	A/Ostrich/South_Africa/S2017/08_0362_P8_34/2017	National Institute for Communicable Diseases
EPI_ISL_285618	A/Ostrich/South_Africa/S2017/08_0362_P7/2017	National Institute for Communicable Diseases
EPI_ISL_285608	A/Ostrich/South_Africa/S2017/08_0362_P8_33/2017	National Institute for Communicable Diseases
EPI_ISL_369343	A/chicken/South_Africa/441839/2017	National Institute for Communicable Diseases
EPI_ISL_369339	A/chicken/South_Africa/440638A/2017	National Institute for Communicable Diseases
EPI_ISL_369340	A/chicken/South_Africa/440638B/2017	National Institute for Communicable Diseases
EPI_ISL_369336	A/chicken/South_Africa/Villiers/2017	National Institute for Communicable Diseases
EPI_ISL_369342	A/chicken/South_Africa/MC002/2017	National Institute for Communicable Diseases
EPI_ISL_369338	A/chicken/South_Africa/436893/2017	National Institute for Communicable Diseases
EPI_ISL_381811	A/Speckled_pigeon/South_Africa/08-004B/2017	National Institute for Communicable Diseases
EPI_ISL_369363	A/chicken/South_Africa/115370/2017	National Institute for Communicable Diseases
EPI_ISL_285653	A/Pigeon/South_Africa/S2017/08_0323_P1/2017	National Institute for Communicable Diseases
EPI_ISL_285604	A/Ostrich/South_Africa/S2017/08_0046_P3/2017	National Institute for Communicable Diseases
EPI_ISL_285917	A/Chicken/South_Africa/S2017/09_0184_62/2017	National Institute for Communicable Diseases
EPI_ISL_285625	A/Duck/South_Africa/S2017/08_0481_P2/2017	National Institute for Communicable Diseases
EPI_ISL_285944	A/Chicken/South_Africa/S2017/08_0336_P2/2017	National Institute for Communicable Diseases
EPI_ISL_285480	A/Ostrich/South_Africa/S2017/08_0362_P10/2017	National Institute for Communicable Diseases
EPI_ISL_285512	A/Chicken/South_Africa/S2017/08_0336_P1/2017	National Institute for Communicable Diseases
EPI_ISL_285616	A/Ostrich/South_Africa/S2017/08_0161_P8/2017	National Institute for Communicable Diseases
EPI_ISL_369345	A/chicken/South_Africa/443397/2017	National Institute for Communicable Diseases
EPI_ISL_237554	A/painted_stork/India/10CA03/2016	ICAR-National Institute of High Security Animal Diseases
EPI_ISL_240677	A/domestic_duck/Siberia/103/2016	Research Institute of Experimental and Clinical Medicine
EPI_ISL_250231	A/domestic_duck/Siberia/49_feather/2016	Research Institute of Experimental and Clinical Medicine
EPI_ISL_240678	A/domestic_duck/Siberia/50K/2016	Research Institute of Experimental and Clinical Medicine
EPI_ISL_297466	A/chicken/Kostroma/1721/2017	State Research Center of Virology and Biotechnology
EPI_ISL_297465	A/chicken/Kostroma/1720/2017	State Research Center of Virology and Biotechnology
EPI_ISL_297464	A/chicken/Kostroma/1719/2017	State Research Center of Virology and Biotechnology
EPI_ISL_297463	A/chicken/Kostroma/1717/2017	State Research Center of Virology and Biotechnology
EPI_ISL_295027	A/chicken/Kostroma/1718/2017	State Research Center of Virology and Biotechnology

EPI_ISL_254745	A/mallard_duck/Korea/WA137/2017	Animal and Plant Quarantine Agency (S-2026)
EPI_ISL_254725	A/chicken/Korea/H903/2017	Animal and Plant Quarantine Agency (S-2026)
EPI_ISL_292498	A/Bar-headed_Goose/Qinghai/a210/2017	Wuhan Institute of Virology
EPI_ISL_292479	A/Bar-headed_Goose/Qinghai/a893/2017	Wuhan Institute of Virology
EPI_ISL_292478	A/Bar-headed_Goose/Qinghai/a765/2017	Wuhan Institute of Virology
EPI_ISL_292477	A/Great_Crested_Grebe/Qinghai/a737/2017	Wuhan Institute of Virology
EPI_ISL_292476	A/Bar-headed_Goose/Qinghai/a237/2017	Wuhan Institute of Virology
EPI_ISL_292335	A/water/Qinghai/XXII2871/2017	Wuhan Institute of Virology
EPI_ISL_292334	A/Bar-headed_Goose/Qinghai/B655/2017	Wuhan Institute of Virology
EPI_ISL_292341	A/Bar-headed_Goose/Qinghai/a218/2017	Wuhan Institute of Virology
EPI_ISL_292340	A/Bar-headed_Goose/Qinghai/a230/2017	Wuhan Institute of Virology
EPI_ISL_306958	A/Ornamental_bird/Al-Qasim/AI9/2017	Wuhan Institute of Virology
EPI_ISL_306956	A/Falcon/Riyadh/AI5/2017	Center of Influenza Research, University of Hong Kong
EPI_ISL_306955	A/Bulbul/Riyadh/AI4/2017	Center of Influenza Research, University of Hong Kong
EPI_ISL_306915	A/Chicken/Riyadh/A15/2018	Center of Influenza Research, University of Hong Kong
EPI_ISL_306912	A/Chicken/Riyadh/AI6/2017	Center of Influenza Research, University of Hong Kong
EPI_ISL_306910	A/Turkey/Riyadh/AI1/2017	Center of Influenza Research, University of Hong Kong
EPI_ISL_306914	A/Chicken/Riyadh/AI10/2017	Center of Influenza Research, University of Hong Kong
EPI_ISL_306959	A/Holland_pigeon/Riyadh/AI3/2017	Center of Influenza Research, University of Hong Kong
EPI_ISL_306957	A/Chicken/Al-Ahsaa/AI8/2017	Center of Influenza Research, University of Hong Kong
EPI_ISL_306913	A/Chicken/Al-Ahsaa/AI7/2017	Center of Influenza Research, University of Hong Kong
EPI_ISL_306911	A/Duck/Riyadh/AI2/2017	Center of Influenza Research, University of Hong Kong
EPI_ISL_231685	A/black-headed_gull/Tyva/41/2016	WHO National Influenza Centre Russian Federation
EPI_ISL_231684	A/wild_duck/Tyva/35/2016	WHO National Influenza Centre Russian Federation
EPI_ISL_230820	A/great_crested_grebe/Tyva/34/2016	WHO National Influenza Centre Russian Federation
EPI_ISL_240109	A/chicken/Kalmykia/2661/2016	State Research Center of Virology and Biotechnology
EPI_ISL_248666	A/bronze_turkey/Czech_Republic/1414-17/2017	State Veterinary Institute Prague
EPI_ISL_247724	A/wild_duck/Tatarstan/3059/2016	State Research Center of Virology and Biotechnology
EPI_ISL_297234	A/chicken/Rostov-on-Don/1321/2017	State Research Center of Virology and Biotechnology
EPI_ISL_237965	A/goose/Hungary/55128/2016	Central Agricultural Office Veterinary Diagnostic Directorate
EPI_ISL_239069	A/mute_swan/Croatia/85/2016	Croatian Veterinary Institute
EPI_ISL_237731	A/domestic_turkey/Hungary/53433/2016	Central Agricultural Office Veterinary Diagnostic Directorate
EPI_ISL_256460	A/Turkey/Hungary/53136/2016	Central Agricultural Office Veterinary Diagnostic Directorate
EPI_ISL_238197	A/mute_swan/Croatia/78/2016	Croatian Veterinary Institute
EPI_ISL_255193	A/Duck/Hungary/984/2017	Central Agricultural Office Veterinary Diagnostic Directorate
EPI_ISL_378256	A/Mulard_Duck/Hungary/59163/2016	Central Agricultural Office Veterinary Diagnostic Directorate
EPI_ISL_271714	A/Mulard_duck/Hungary/59163/2016	Central Agricultural Office Veterinary Diagnostic Directorate
EPI_ISL_255935	A/Mute_swan/Hungary/6276/2017	Central Agricultural Office Veterinary Diagnostic Directorate
EPI_ISL_239420	A/duck/Hungary/60441/2016	Central Agricultural Office Veterinary Diagnostic Directorate
EPI_ISL_271711	A/Mulard_duck/Hungary/62902/2016	Central Agricultural Office Veterinary Diagnostic Directorate
EPI_ISL_271710	A/Mulard_duck/Hungary/60369/2016	Central Agricultural Office Veterinary Diagnostic Directorate
EPI_ISL_271708	A/Goose/Hungary/17985/2017	Central Agricultural Office Veterinary Diagnostic Directorate
EPI_ISL_271706	A/Goose/Hungary/17051/2017	Central Agricultural Office Veterinary Diagnostic Directorate
EPI_ISL_271705	A/Goose/Hungary/15729/2017	Central Agricultural Office Veterinary Diagnostic Directorate
EPI_ISL_271704	A/Goose/Hungary/17261/2017	Central Agricultural Office Veterinary Diagnostic Directorate

EPI_ISL_256453	A/Duck/Hungary/54738/2016	Central Agricultural Office Veterinary Diagnostic Directorate
EPI_ISL_255191	A/Harris_Hawk/Hungary/120/2017	Central Agricultural Office Veterinary Diagnostic Directorate
EPI_ISL_237964	A/Mulard_duck/Hungary/54494/2016	Central Agricultural Office Veterinary Diagnostic Directorate
EPI_ISL_271713	A/Goose/Hungary/64909/2016	Central Agricultural Office Veterinary Diagnostic Directorate
EPI_ISL_271712	A/Goose/Hungary/63743/2016	Central Agricultural Office Veterinary Diagnostic Directorate
EPI_ISL_271709	A/Goose/Hungary/59763/2016	Central Agricultural Office Veterinary Diagnostic Directorate
EPI_ISL_271707	A/Goose/Hungary/17580/2017	Central Agricultural Office Veterinary Diagnostic Directorate
EPI_ISL_255937	A/Pheasant/Hungary/7685/2017	Central Agricultural Office Veterinary Diagnostic Directorate
EPI_ISL_255936	A/Pheasant/Hungary/6553/2017	Central Agricultural Office Veterinary Diagnostic Directorate
EPI_ISL_237966	A/duck/Hungary/55191/2016	Central Agricultural Office Veterinary Diagnostic Directorate
EPI_ISL_239419	A/chicken/Hungary/59048/2016	Central Agricultural Office Veterinary Diagnostic Directorate
EPI_ISL_255938	A/Rook/Hungary/4975/2017	Central Agricultural Office Veterinary Diagnostic Directorate
EPI_ISL_256457	A/Duck/Hungary/55764/2016	Central Agricultural Office Veterinary Diagnostic Directorate
EPI_ISL_255398	A/Goose/Hungary/59712/2016	Central Agricultural Office Veterinary Diagnostic Directorate
EPI_ISL_237730	A/mute_swan/Hungary/51049/2016	Central Agricultural Office Veterinary Diagnostic Directorate
EPI_ISL_255212	A/Greylag_goose/Hungary/320/2017	Central Agricultural Office Veterinary Diagnostic Directorate
EPI_ISL_255220	A/Mallard/Hungary/1574a/2017	Central Agricultural Office Veterinary Diagnostic Directorate
EPI_ISL_255218	A/Mallard/Hungary/1574b/2017	Central Agricultural Office Veterinary Diagnostic Directorate
EPI_ISL_297235	A/chicken/Rostov-on-Don/1598/2017	State Research Center of Virology and Biotechnology
EPI_ISL_268652	A/Eur_Wig/NL-Zoeterwoude/16015702-010/2016	Wageningen Bioveterinary Research
EPI_ISL_268645	A/Eur_Wig/NL-Leidschendam/16015697-007/2016	Wageningen Bioveterinary Research
EPI_ISL_268641	A/Eur_Wig/NL-Gouda/16015824-001/2016	Wageningen Bioveterinary Research
EPI_ISL_268637	A/Eur_Wig/NL-De_Waal_(Texel)/16014891-004/2016	Wageningen Bioveterinary Research
EPI_ISL_268636	A/Eur_Wig/NL-De_Waal_(Texel)/16014891-003/2016	Wageningen Bioveterinary Research
EPI_ISL_268657	A/Grey_Go/NL-Groot-Ammers/16015901-012/2016	Wageningen Bioveterinary Research
EPI_ISL_268653	A/Eur_Wig/NL-Zwolle/16015820-002/2016	Wageningen Bioveterinary Research
EPI_ISL_268626	A/Ch/NL-Rhenen/16016141-006/2016	Wageningen Bioveterinary Research
EPI_ISL_268650	A/Eur_Wig/NL-West_Graftdijk/16015746-003/2016	Wageningen Bioveterinary Research
EPI_ISL_268648	A/Eur_Wig/NL-Vianen/16015917-006/2016	Wageningen Bioveterinary Research
EPI_ISL_268646	A/Eur_Wig/NL-Reeuwijk/16015903-003/2016	Wageningen Bioveterinary Research
EPI_ISL_268632	A/Dk/NL-Kamperveen/16016104-001-005/2016	Wageningen Bioveterinary Research
EPI_ISL_239801	A/turkey/England/052131/2016	Animal and Plant Health Agency (APHA)
EPI_ISL_268627	A/Ch/NL-Zoeterwoude/16016484-021-025/2016	Wageningen Bioveterinary Research
EPI_ISL_268623	A/Ch/NL-Boven_Leeuwen/16016151-006-010/2016	Wageningen Bioveterinary Research
EPI_ISL_268642	A/Eur_Wig/NL-Greonterp/16015653-001/2016	Wageningen Bioveterinary Research
EPI_ISL_275287	A/chicken/Rostov-on-Don/44/2017	State Research Center of Virology and Biotechnology
EPI_ISL_247723	A/mute_swan/Krasnodar/25/2017	State Research Center of Virology and Biotechnology
EPI_ISL_268651	A/Eur_Wig/NL-Wormer/16016143-002/2016	Wageningen Bioveterinary Research
EPI_ISL_268649	A/Eur_Wig/NL-Walterswald/16015923-003/2016	Wageningen Bioveterinary Research
EPI_ISL_268638	A/Eur_Wig/NL-Drieborg_(Dollard)/16015513-001/2016	Wageningen Bioveterinary Research
EPI_ISL_339105	A/Duck/France/RG1/2016_(H5N8)	Ecole Veterinaire de Toulouse
EPI_ISL_255934	A/Mute_swan/Hungary/6092/2017	Central Agricultural Office Veterinary Diagnostic Directorate
EPI_ISL_255200	A/Mute_swan/Hungary/2508/2017	Central Agricultural Office Veterinary Diagnostic Directorate
EPI_ISL_224580	A/great_crested_grebe/Uvs-Nuur_Lake/341/2016	Research Institute of Experimental and Clinical Medicine
EPI_ISL_268625	A/Ch/NL-Hiaure/16016112-001-005/2016	Wageningen Bioveterinary Research

EPI_ISL_333615	A/gadwall/Chany/893/2018	State Research Center of Virology and Biotechnology
EPI_ISL_287564	A/T_Dk/NL-Werkendam/16014159-001/2016	Wageningen Bioveterinary Research
EPI_ISL_268672	A/T_Dk/NL-Werkendam/16014159-002/2016	Wageningen Bioveterinary Research
EPI_ISL_268671	A/T_Dk/NL-Rotterdam/16014155-001/2016	Wageningen Bioveterinary Research
EPI_ISL_268670	A/T_Dk/NL-Roggebotsluis/16014462-015/2016	Wageningen Bioveterinary Research
EPI_ISL_268669	A/T_Dk/NL-Monnickendam/16013865-006-008/2016	Wageningen Bioveterinary Research
EPI_ISL_268666	A/P_falcon/NL-Vrouwenpolder_(Zeeland)/16015510-001/2016	Wageningen Bioveterinary Research
EPI_ISL_268660	A/Gull10/NL-Marker_Wadden/16014466-014/2016	Wageningen Bioveterinary Research
EPI_ISL_268659	A/Gull1/NL-Marker_Wadden/16014466-011/2016	Wageningen Bioveterinary Research
EPI_ISL_268658	A/Gull/NL-Marker_Wadden/16014466-020/2016	Wageningen Bioveterinary Research
EPI_ISL_268656	A/Gr_bk_bd_gull/NL-Slootdorp/16014102-005/2016	Wageningen Bioveterinary Research
EPI_ISL_268655	A/Go/NL-Roggebotsluis/16014462-010/2016	Wageningen Bioveterinary Research
EPI_ISL_268654	A/G_c_grebe/NL-Monnickendam/16013865-009-010/2016	Wageningen Bioveterinary Research
EPI_ISL_268633	A/Dk/NL-Rotterdam/16014008-001-005/2016	Wageningen Bioveterinary Research
EPI_ISL_268624	A/Ch/NL-Den_Oever/16014231-001/2016	Wageningen Bioveterinary Research
EPI_ISL_268621	A/C_Gull/NL-Slootdorp/16014102-003/2016	Wageningen Bioveterinary Research
EPI_ISL_268619	A/Bl_H_gull/NL-Slootdorp/16014102-002/2016	Wageningen Bioveterinary Research
EPI_ISL_274858	A/mute_swan/Kaliningrad/132/2017	Wageningen Bioveterinary Research
EPI_ISL_268678	A/T_Dk/NL-Zeewolde/16013976-005/2016	Wageningen Bioveterinary Research
EPI_ISL_268677	A/T_Dk/NL-Zeewolde/16013976-004-006/2016	Wageningen Bioveterinary Research
EPI_ISL_268665	A/Mal/NL-Mastenbroek/16015378-002/2016	Wageningen Bioveterinary Research
EPI_ISL_268639	A/Eur_Wig/NL-Enumatil-Groningen/16015704-001/2016	Wageningen Bioveterinary Research
EPI_ISL_268643	A/Eur_Wig/NL-Groningen/16015376-003/2016	Wageningen Bioveterinary Research
EPI_ISL_268663	A/Magpie/NL-Volendam/16014331-002/2016	Wageningen Bioveterinary Research
EPI_ISL_268661	A/L-bl-ba-gull/NL-Sovon/16014324-014/2016	Wageningen Bioveterinary Research
EPI_ISL_268620	A/Buzzard/NL-Durgerdam/16015100-004/2016	Wageningen Bioveterinary Research
EPI_ISL_268628	A/Crow/NL-Oostwoud/16015372-004/2016	Wageningen Bioveterinary Research
EPI_ISL_268662	A/M_Swan/NL-Roggebotsluis/16014462-019/2016	Wageningen Bioveterinary Research
EPI_ISL_255209	A/Common_tern/Hungary/8187/2017	Central Agricultural Office Veterinary Diagnostic Directorate
EPI_ISL_268681	A/T_Dk/NL-Zuidoost_Beemster/16014148-009/2016	Wageningen Bioveterinary Research
EPI_ISL_268680	A/T_Dk/NL-Zuidoost_Beemster/16014148-002/2016	Wageningen Bioveterinary Research
EPI_ISL_268668	A/T_Dk/NL-Almeerder_Zand/16014341-003/2016	Wageningen Bioveterinary Research
EPI_ISL_268679	A/T_Dk/NL-Zeewolde/16013976-006/2016	Wageningen Bioveterinary Research
EPI_ISL_268676	A/T_Dk/NL-Zeewolde/16013976-004/2016	Wageningen Bioveterinary Research
EPI_ISL_268675	A/T_Dk/NL-Zeewolde/16013976-001-003/2016	Wageningen Bioveterinary Research
EPI_ISL_268674	A/T_Dk/NL-Zeewolde/16013976-001/2016	Wageningen Bioveterinary Research
EPI_ISL_268673	A/T_Dk/NL-Werkendam/16014159-003/2016	Wageningen Bioveterinary Research
EPI_ISL_268618	A/Bk_swan/NL-Den_Oever/16013973-002/2016	Wageningen Bioveterinary Research
EPI_ISL_255910	A/Mew_Gull/Netherlands/1/2016	Erasmus Medical Center
EPI_ISL_268631	A/Dk/NL-Biddinghuizen/16015145-021-025/2016	Wageningen Bioveterinary Research
EPI_ISL_268630	A/Dk/NL-Biddinghuizen/16015083-016-020/2016	Wageningen Bioveterinary Research
EPI_ISL_268629	A/Dk/NL-Biddinghuizen/16014829-011-015/2016	Wageningen Bioveterinary Research
EPI_ISL_268644	A/Eur_Wig/NL-Leeuwarden/16015699-002/2016	Wageningen Bioveterinary Research
EPI_ISL_268682	A/Teal/NL-Ferwert/16015273-013/2016	Wageningen Bioveterinary Research
EPI_ISL_288363	A/chicken/Greece/39_2017a/2017	Thessalonica Veterinary Centre

EPI_ISL_288362	A/chicken/Greece/39_2017/2017	Thessalonica Veterinary Centre
EPI_ISL_256298	A/gadwall/Kurgan/2442/2016	State Research Center of Virology and Biotechnology
EPI_ISL_247725	A/chicken/Kalmykia/2643/2016	State Research Center of Virology and Biotechnology
EPI_ISL_247720	A/chicken/Voronezh/20/2017	State Research Center of Virology and Biotechnology
EPI_ISL_247719	A/chicken/Voronezh/19/2017	State Research Center of Virology and Biotechnology
EPI_ISL_247718	A/chicken/Voronezh/18/2017	State Research Center of Virology and Biotechnology
EPI_ISL_247717	A/long-eared_owl/Voronezh/16/2017	State Research Center of Virology and Biotechnology
EPI_ISL_247716	A/Ural_owl/Voronezh/14/2017	State Research Center of Virology and Biotechnology
EPI_ISL_247715	A/long-eared_owl/Voronezh/15/2017	State Research Center of Virology and Biotechnology
EPI_ISL_282143	A/goose/Italy/17VIR6358-3/2017	Istituto Zooprofilattico Sperimentale Delle Venezie
EPI_ISL_282141	A/swan/Italy/17VIR7064-1/2017	Istituto Zooprofilattico Sperimentale Delle Venezie
EPI_ISL_247722	A/goose/Krasnodar/3144/2017	State Research Center of Virology and Biotechnology
EPI_ISL_255206	A/Peregrine_falcon/Hungary/4882/2017	Central Agricultural Office Veterinary Diagnostic Directorate
EPI_ISL_335457	A/mute_swan/Shimane/3211A002/2017	National Institute of Animal Health
EPI_ISL_268667	A/Sea_eagle/NL-Assen/16015398-002/2016	Wageningen Bioveterinary Research
EPI_ISL_267376	A/mallard_duck/Netherlands/18/2012	Erasmus Medical Center
EPI_ISL_373081	A/Mallard/Netherlands/21/2013	Erasmus Medical Center
EPI_ISL_267224	A/mallard_duck/Netherlands/16/2012	Erasmus Medical Center
EPI_ISL_267243	A/barnacle_goose/Netherlands/2/2014	Erasmus Medical Center
EPI_ISL_243650	A/mallard_duck/Netherlands/24/2009	Erasmus Medical Center
EPI_ISL_309840	A/Chicken/Netherlands/12002495-006-010/2012	Wageningen Bioveterinary Research
EPI_ISL_309930	A/Chicken/Netherlands/14016059/2014	Wageningen Bioveterinary Research
EPI_ISL_309835	A/Duck/Netherlands/14016396/2014	Wageningen Bioveterinary Research
EPI_ISL_309833	A/Duck/Netherlands/14015610/2014	Wageningen Bioveterinary Research
EPI_ISL_332682	A/teal/Toguchin/1157/2016	State Research Center of Virology and Biotechnology
EPI_ISL_332681	A/teal/Toguchin/1156/2016	State Research Center of Virology and Biotechnology
EPI_ISL_332679	A/mallard/Toguchin/1154/2016	State Research Center of Virology and Biotechnology
EPI_ISL_332677	A/teal/Toguchin/1153/2016	State Research Center of Virology and Biotechnology
EPI_ISL_237553	A/duck/India/10CA01/2016	ICAR-National Institute of High Security Animal Diseases
EPI_ISL_292194	A/Bar-headed_Goose/Qinghai/a88/2016	Wuhan Institute of Virology
EPI_ISL_292331	A/Bar-headed_Goose/Qinghai/a114/2016	Wuhan Institute of Virology
EPI_ISL_266820	A/Bean_goose/Hubei/CH-i122/2017_H5N8	Wuhan Institute of Virology
EPI_ISL_266824	A/Bean_goose/Hubei/CH-i320/2017_H5N8	Wuhan Institute of Virology
EPI_ISL_266821	A/Bean_goose/Hubei/CH-i119/2017	Wuhan Institute of Virology
EPI_ISL_240110	A/chicken/Astrakhan/3131/2016	State Research Center of Virology and Biotechnology
EPI_ISL_255219	A/White_fronted_goose/Hungary/801/2017	Central Agricultural Office Veterinary Diagnostic Directorate
EPI_ISL_255215	A/Mute_swan/Hungary/3137/2017	Central Agricultural Office Veterinary Diagnostic Directorate
EPI_ISL_255213	A/GuineaFowl/Hungary/596/2017	Central Agricultural Office Veterinary Diagnostic Directorate
EPI_ISL_255202	A/Mute_swan/Hungary/3139/2017	Central Agricultural Office Veterinary Diagnostic Directorate
EPI_ISL_255196	A/Greylag_goose/Hungary/1941/2017	Central Agricultural Office Veterinary Diagnostic Directorate
EPI_ISL_275433	A/unknown/Tatarstan/94/2017	State Research Center of Virology and Biotechnology
EPI_ISL_275288	A/chicken/Tatarstan/88/2017	State Research Center of Virology and Biotechnology
EPI_ISL_275283	A/chicken/Shchyolkovo/47/2017	State Research Center of Virology and Biotechnology
EPI_ISL_256300	A/chicken/Sergiyev_Posad/39/2017	State Research Center of Virology and Biotechnology
EPI_ISL_256299	A/chicken/Sergiyev_Posad/38/2017	State Research Center of Virology and Biotechnology

EPI_ISL_275432	A/unknown/Tatarstan/86/2017	State Research Center of Virology and Biotechnology
EPI_ISL_322984	A/Grey_seal/361-10/BalticPL/16	Stiftung Tierärztliche Hochschule Hannover
EPI_ISL_257699	A/Tufted_Duck/Switzerland/V237/2016	Faculty of Veterinary Medicine at the University of Bern
EPI_ISL_240012	A/duck/France/161108h/2016	Agence Nationale De Securite Sanitaire De L'alimentation
EPI_ISL_255933	A/Cormorant/Hungary/6102/2017	Central Agricultural Office Veterinary Diagnostic Directorate
EPI_ISL_256462	A/Mute_swan/Hungary/5879/2017	Central Agricultural Office Veterinary Diagnostic Directorate
EPI_ISL_288411	A/Go/NL-Utrecht/17006881-001/2017	Wageningen Bioveterinary Research
EPI_ISL_287565	A/M_Swan/NL-Groningen/16015826-001/2016	Wageningen Bioveterinary Research
EPI_ISL_337151	A/chicken/Mari_El/870/2018	State Research Center of Virology and Biotechnology
EPI_ISL_320955	A/chicken/Cheboksary/851/2018	State Research Center of Virology and Biotechnology
EPI_ISL_320954	A/chicken/Cheboksary/850/2018	State Research Center of Virology and Biotechnology
EPI_ISL_320953	A/chicken/Cheboksary/849/2018	State Research Center of Virology and Biotechnology
EPI_ISL_320685	A/chicken/Samara/679/2018	State Research Center of Virology and Biotechnology
EPI_ISL_320684	A/goose/Samara/675/2018	State Research Center of Virology and Biotechnology
EPI_ISL_320683	A/goose/Samara/673/2018	State Research Center of Virology and Biotechnology
EPI_ISL_320682	A/chicken/Kursk/762/2018	State Research Center of Virology and Biotechnology
EPI_ISL_288439	A/chicken/Korea/Gimje2/2017	Animal and Plant Quarantine Agency (S-2145)
EPI_ISL_292187	A/Bar-headed_Goose/Qinghai/XX782/2016	Wuhan Institute of Virology
EPI_ISL_292170	A/Bar-headed_Goose/Qinghai/A22/2016	Wuhan Institute of Virology
EPI_ISL_292495	A/Bar-headed_Goose/Qinghai/B11/2016	Wuhan Institute of Virology
EPI_ISL_292181	A/Bar-headed_Goose/Qinghai/A19/2016	Wuhan Institute of Virology
EPI_ISL_292179	A/Bar-headed_Goose/Qinghai/XX13/2016	Wuhan Institute of Virology
EPI_ISL_292332	A/water/Qinghai/i34-39/2016	Wuhan Institute of Virology
EPI_ISL_292175	A/Bar-headed_Goose/Qinghai/XX446/2016	Wuhan Institute of Virology
EPI_ISL_266822	A/Herring_Gull/Hubei/CH-i149/2017_H5N8	Wuhan Institute of Virology
EPI_ISL_292333	A/Water/Qinghai/i40-44/2016	Wuhan Institute of Virology
EPI_ISL_255214	A/Chicken/Hungary/1751/2017	Central Agricultural Office Veterinary Diagnostic Directorate
EPI_ISL_254814	A/mallard/Hungary/57857/2016	Central Agricultural Office Veterinary Diagnostic Directorate
EPI_ISL_255216	A/Mallard/Hungary/5821/2017	Central Agricultural Office Veterinary Diagnostic Directorate
EPI_ISL_255192	A/Goose/Hungary/982/2017	Central Agricultural Office Veterinary Diagnostic Directorate
EPI_ISL_255198	A/Turkey/Hungary/2030/2017	Central Agricultural Office Veterinary Diagnostic Directorate
EPI_ISL_255190	A/Mute_swan/Hungary/119/2017	Central Agricultural Office Veterinary Diagnostic Directorate
EPI_ISL_255195	A/Duck/Hungary/1588/2017	Central Agricultural Office Veterinary Diagnostic Directorate
EPI_ISL_255174	A/Goose/Hungary/65817/2016	Central Agricultural Office Veterinary Diagnostic Directorate
EPI_ISL_247721	A/turkey/Rostov-on-Don/11/2017	State Research Center of Virology and Biotechnology
EPI_ISL_268622	A/Ch/NL-Abbega/X16015736/2016	Wageningen Bioveterinary Research
EPI_ISL_268664	A/Mal/NL-IJsselmuiden/16015448-002/2016	Wageningen Bioveterinary Research
EPI_ISL_253036	A/turkey/England/003778/2017	Animal and Plant Health Agency (APHA)
EPI_ISL_253037	A/chicken/Wales/000023/2016	Animal and Plant Health Agency (APHA)
EPI_ISL_268634	A/Dk/NL-Stolwijk/16016291-016-020/2016	Wageningen Bioveterinary Research
EPI_ISL_255182	A/turkey/Italy/17VIR538-1/2017	Istituto Zooprofilattico Sperimentale Delle Venezie
EPI_ISL_288438	A/chicken/Korea/Gunsan/2017	Animal and Plant Quarantine Agency (S-2145)
EPI_ISL_333629	A/Environment/Fujiansanyuan/08/2017	Fujian Center for Disease Control and Prevention
EPI_ISL_256301	A/environment/Kamchatka/18/2016	State Research Center of Virology and Biotechnology
EPI_ISL_292326	A/Great_Cormorant/Qinghai/a51/2016	Wuhan Institute of Virology

EPI_ISL_292192	A/Bar-headed_Goose/Qinghai/A23/2016	Wuhan Institute of Virology
EPI_ISL_292191	A/Bar-headed_Goose/Qinghai/B12/2016	Wuhan Institute of Virology
EPI_ISL_292188	A/Great_Cormorant/Qinghai/B82/2016	Wuhan Institute of Virology
EPI_ISL_224744	A/Brown-headed_Gull/Qinghai/ZTO3-B/2016	Wuhan Institute of Virology
EPI_ISL_224743	A/Brown-headed_Gull/Qinghai/ZTO1-LU/2016	Wuhan Institute of Virology
EPI_ISL_224734	A/Bar-headed_Goose/Qinghai/BTY15-LU/2016	Wuhan Institute of Virology
EPI_ISL_224726	A/Bar-headed_Goose/Qinghai/BTY11-LU/2016	Wuhan Institute of Virology
EPI_ISL_224717	A/Bar-headed_Goose/Qinghai/BTY7-LU1/2016	Wuhan Institute of Virology
EPI_ISL_288364	A/chicken/Greece/39_2017b/2017	Thessalonica Veterinary Centre (TVC)
EPI_ISL_292497	A/Bar-headed_Goose/Qinghai/XXI122/2016	Wuhan Institute of Virology
EPI_ISL_292330	A/Bar-headed_Goose/Qinghai/a26/2016	Wuhan Institute of Virology
EPI_ISL_292327	A/Bar-headed_Goose/Qinghai/p2/2016	Wuhan Institute of Virology
EPI_ISL_292237	A/Bar-headed_Goose/Qinghai/a15/2016	Wuhan Institute of Virology
EPI_ISL_292231	A/Bar-headed_Goose/Qinghai/a61/2016	Wuhan Institute of Virology
EPI_ISL_292190	A/Bar-headed_Goose/Qinghai/XX111/2016	Wuhan Institute of Virology
EPI_ISL_292180	A/Bar-headed_Goose/Qinghai/XX22/2016	Wuhan Institute of Virology
EPI_ISL_224729	A/Bar-headed_Goose/Qinghai/BTY13-B/2016	Wuhan Institute of Virology
EPI_ISL_224715	A/Bar-headed_Goose/Qinghai/BTY6-LU/2016	Wuhan Institute of Virology
EPI_ISL_292229	A/Bar-headed_Goose/Qinghai/a113/2016	Wuhan Institute of Virology
EPI_ISL_292184	A/Bar-headed_Goose/Qinghai/B51/2016	Wuhan Institute of Virology
EPI_ISL_292174	A/Bar-headed_Goose/Qinghai/XX431/2016	Wuhan Institute of Virology
EPI_ISL_292176	A/Bar-headed_Goose/Qinghai/A17/2016	Wuhan Institute of Virology
EPI_ISL_292235	A/Bar-headed_Goose/Qinghai/a43/2016	Wuhan Institute of Virology
EPI_ISL_292232	A/Bar-headed_Goose/Qinghai/HDT001/2016	Wuhan Institute of Virology
EPI_ISL_292178	A/Bar-headed_Goose/Qinghai/A12/2016	Wuhan Institute of Virology
EPI_ISL_224751	A/Brown-headed_Gull/Qinghai/ZTO6-MU/2016	Wuhan Institute of Virology
EPI_ISL_224746	A/Brown-headed_Gull/Qinghai/ZTO4-B/2016	Wuhan Institute of Virology
EPI_ISL_224733	A/Bar-headed_Goose/Qinghai/BTY15-B/2016	Wuhan Institute of Virology
EPI_ISL_224731	A/Bar-headed_Goose/Qinghai/BTY14-B/2016	Wuhan Institute of Virology
EPI_ISL_224730	A/Bar-headed_Goose/Qinghai/BTY13-LU/2016	Wuhan Institute of Virology
EPI_ISL_224716	A/Bar-headed_Goose/Qinghai/BTY7-B/2016	Wuhan Institute of Virology
EPI_ISL_224725	A/Bar-headed_Goose/Qinghai/BTY11-B/2016	Wuhan Institute of Virology
EPI_ISL_224735	A/Bar-headed_Goose/Qinghai/BTY16-B/2016	Wuhan Institute of Virology
EPI_ISL_224742	A/Brown-headed_Gull/Qinghai/ZTO1-B/2016	Wuhan Institute of Virology
EPI_ISL_291952	A/Bar-headed_Goose/Qinghai/B44/2016	Wuhan Institute of Virology
EPI_ISL_292183	A/Bar-headed_Goose/Qinghai/A13/2016	Wuhan Institute of Virology
EPI_ISL_292328	A/Bar-headed_Goose/Qinghai/a45/2016	Wuhan Institute of Virology
EPI_ISL_292329	A/Bar-headed_Goose/Qinghai/a27/2016	Wuhan Institute of Virology
EPI_ISL_292236	A/Bar-headed_Goose/Qinghai/a24/2016	Wuhan Institute of Virology
EPI_ISL_292193	A/Bar-headed_Goose/Qinghai/XX76/2016	Wuhan Institute of Virology
EPI_ISL_224724	A/Bar-headed_Goose/Qinghai/BTY10-LU/2016	Wuhan Institute of Virology
EPI_ISL_224714	A/Bar-headed_Goose/Qinghai/BTY6-B/2016	Wuhan Institute of Virology
EPI_ISL_224707	A/Bar-headed_Goose/Qinghai/BTY2-B/2016	Wuhan Institute of Virology
EPI_ISL_224708	A/Bar-headed_Goose/Qinghai/BTY2-LU/2016	Wuhan Institute of Virology
EPI_ISL_292230	A/Bar-headed_Goose/Qinghai/a93/2016	Wuhan Institute of Virology

EPI_ISL_224745	A/Brown-headed_Gull/Qinghai/ZTO3-LU/2016	Wuhan Institute of Virology
EPI_ISL_224750	A/Brown-headed_Gull/Qinghai/ZTO6-SP/2016	Wuhan Institute of Virology
EPI_ISL_224749	A/Brown-headed_Gull/Qinghai/ZTO6-B/2016	Wuhan Institute of Virology
EPI_ISL_224736	A/Bar-headed_Goose/Qinghai/BTY16-LU/2016	Wuhan Institute of Virology
EPI_ISL_224741	A/Bar-headed_Goose/Qinghai/BTY18-MU/2016	Wuhan Institute of Virology
EPI_ISL_224732	A/Bar-headed_Goose/Qinghai/BTY14-LU/2016	Wuhan Institute of Virology
EPI_ISL_224740	A/Bar-headed_Goose/Qinghai/BTY18-LU/2016	Wuhan Institute of Virology
EPI_ISL_224739	A/Bar-headed_Goose/Qinghai/BTY18-B/2016	Wuhan Institute of Virology
EPI_ISL_224723	A/Bar-headed_Goose/Qinghai/BTY10-B/2016	Wuhan Institute of Virology
EPI_ISL_292177	A/Bar-headed_Goose/Qinghai/A20/2016	Wuhan Institute of Virology
EPI_ISL_292182	A/Bar-headed_Goose/Qinghai/A16/2016	Wuhan Institute of Virology
EPI_ISL_292185	A/Great_Cormorant/Qinghai/Y01/2016	Wuhan Institute of Virology
EPI_ISL_224718	A/Bar-headed_Goose/Qinghai/BTY7-LU2/2016	Wuhan Institute of Virology
EPI_ISL_224747	A/Brown-headed_Gull/Qinghai/ZTO5-B/2016	Wuhan Institute of Virology
EPI_ISL_224728	A/Bar-headed_Goose/Qinghai/BTY12-LU/2016	Wuhan Institute of Virology
EPI_ISL_224727	A/Bar-headed_Goose/Qinghai/BTY12-B/2016	Wuhan Institute of Virology
EPI_ISL_292238	A/Bar-headed_Goose/Qinghai/p18/2016	Wuhan Institute of Virology
EPI_ISL_292233	A/Bar-headed_Goose/Qinghai/a91/2016	Wuhan Institute of Virology
EPI_ISL_292173	A/Bar-headed_Goose/Qinghai/B54/2016	Wuhan Institute of Virology
EPI_ISL_224720	A/Bar-headed_Goose/Qinghai/BTY8-LU/2016	Wuhan Institute of Virology
EPI_ISL_224719	A/Bar-headed_Goose/Qinghai/BTY8-B/2016	Wuhan Institute of Virology
EPI_ISL_224712	A/Bar-headed_Goose/Qinghai/BTY4-LU/2016	Wuhan Institute of Virology
EPI_ISL_224704	A/Bar-headed_Goose/Qinghai/BTY1-B/2016	Wuhan Institute of Virology
EPI_ISL_224748	A/Brown-headed_Gull/Qinghai/ZTO5-K/2016	Wuhan Institute of Virology
EPI_ISL_224721	A/Bar-headed_Goose/Qinghai/BTY9-B/2016	Wuhan Institute of Virology
EPI_ISL_224722	A/Bar-headed_Goose/Qinghai/BTY9-LU/2016	Wuhan Institute of Virology
EPI_ISL_292496	A/Bar-headed_Goose/Qinghai/A11/2016	Wuhan Institute of Virology
EPI_ISL_292198	A/Bar-headed_Goose/Qinghai/a115/2016	Wuhan Institute of Virology
EPI_ISL_404993	A/white-fronted Goose/Germany-BB/AI00018/2020	Friedrich-Loeffler-Institute
EPI_ISL_410291	A/chicken/Germany-BW/AI00049/2020	Friedrich-Loeffler-Institute
EPI_ISL_405813	A/hawk/Poland/003/2020	National Veterinary Research Institut Poland
EPI_ISL_402134	A/turkey/Poland/23/2019	National Veterinary Research Institut Poland
EPI_ISL_405391	A/chicken/Czech Republic/1175-1/2020	State Veterinary Institute Prague
EPI_ISL_405278	A/guinea_fowl/Nigeria/OG-GF11T_19VIR8424-7/2019	Istituto Zooprofilattico Sperimentale Delle Venezie
